# High prevalence of the UGT1A1*28 variant in HIV-infected individuals in Greece

**DOI:** 10.1186/1758-2652-13-S4-P146

**Published:** 2010-11-08

**Authors:** P Panagopoulos, D Paraskevis, V Sypsa, M Detsika, K Protopapas, V Sakka, G Poulakou, A Papadopoulos, G Petrikkos, A Hatzakis

**Affiliations:** 1 Attikon University General Hospital, 4th Department of Internal Medicine, Athens, Greece; 2 Medical School, University of Athens, Department of Hygiene, Epidemiology and Medical St, Athens, Greece; 3 Attikon University General Hospital, 1st Rimini Street, Athens, Greece

## Background

Over the past few years there has been a remarkable increase in our knowledge of the variation in human genome. In parallel genotyping technologies have advanced significantly and allow sufficient throughput to accommodate genome-wide approaches. Hyperbilirubinemia is the most common adverse event in patients treated with atazanavir (ATV). Previous studies showed that polymorphisms in the uridine-glucuronosyl transferase (UGT1A1) enzyme and specifically the UGT1A1*28 variant may influence the risk of hyperbilirubinemia in patients treated with ATV/r.

## Purpose of the study

Our objective was to estimate the prevalence of UGT1A1*28 polymorphism in HIV-infected individuals in Greece and to determine its potential association with hyperbilirubinemia in patients receiving boosted ATV (ATV/r).

## Patients and methods

The prevalence of the UGTA1A1*28 variant was estimated in 80 HIV-infected patients retrospectively, (4/2009-5/2010) prior to the administration of the first-line treatment. Wilcoxon rank-sum test was used to determine whether the total bilirubin levels were different among carriers and non-carriers of the UGT1A1*28 polymorphism. The presence of the UGT1A1*28 allele was detected by PCR and DNA electrophoresis.

## Results

The UGTA1A1*28 variant was detected in 45 out of 80 individuals (56.25%). Among 55 patients who received HAART, 20 received ATV/r as part of their first treatment. Of the ATV/r treated patients, 13 were found to be carriers of the UGT1A1*28 variant (65%). Total bilirubin levels were significantly higher in patients harbouring the UGT1A1*28 polymorphism (median value: 5.15 mg/dl) versus those harbouring the wild type UGT1A1 locus (median value mg/dl: 1.30) (p<0.01). The higher value of bilirubin was observed at week 4 of treatment whereas only 3 patients switched ATV/r to other Protease Inhibitor due to aesthetic problems. Hyperbilirubinemia (total bilirubin >1.3 mg/dl) was not detected in any patient with the UGT11A1*28 variant receiving any other therapy than ATV/r based first-line regimens.

## Conclusions

Notably, 56% of the HIV-infected patients from a single HIV Unit in Greece carry at least one copy of the UGT1A1*28 allele. Carriers of the UGT1A1*28 variant treated with ATV/r based regimens had significantly higher levels of total bilirubin than those with UGT1A1 wild type locus, thus, suggesting the clinical utility of the UGT1A1 testing prior the administration of first-line treatment.

